# LAG: Layered Objects to Generate Better Anchors for Object Detection in Aerial Images

**DOI:** 10.3390/s22103891

**Published:** 2022-05-20

**Authors:** Xueqiang Wan, Jiong Yu, Haotian Tan, Junjie Wang

**Affiliations:** 1 School of Software, Xinjiang University, Urumqi 830091, China; yujiong@xju.edu.cn (J.Y.); wangjunjie@stu.xju.edu.cn (J.W.); 2 College of Information Science and Engineering, Xinjiang University, Urumqi 830046, China; tanhaotian@stu.xju.edu.cn

**Keywords:** anchor generation algorithm, object detection, YOLO, aerial images

## Abstract

You Only Look Once (YOLO) series detectors are suitable for aerial image object detection because of their excellent real-time ability and performance. Their high performance depends heavily on the anchor generated by clustering the training set. However, the effectiveness of the general Anchor Generation algorithm is limited by the unique data distribution of the aerial image dataset. The divergence in the distribution of the number of objects with different sizes can cause the anchors to overfit some objects or be assigned to suboptimal layers because anchors of each layer are generated uniformly and affected by the overall data distribution. In this paper, we are inspired by experiments under different anchors settings and proposed the Layered Anchor Generation (LAG) algorithm. In the LAG, objects are layered by their diagonals, and then anchors of each layer are generated by analyzing the diagonals and aspect ratio of objects of the corresponding layer. In this way, anchors of each layer can better match the detection range of each layer. Experiment results showed that our algorithm is of good generality that significantly uprises the performance of You Only Look Once version 3 (YOLOv3), You Only Look Once version 5 (YOLOv5), You Only Learn One Representation (YOLOR), and Cascade Regions with CNN features (Cascade R-CNN) on the Vision Meets Drone (VisDrone) dataset and the object DetectIon in Optical Remote sensing images (DIOR) dataset, and these improvements are cost-free.

## 1. Introduction

A variety of information is hidden in images, and some studies were devoted to extracting the key information from various images [1,2]. Object detection is to find objects that we want to pay attention to in images and then locate their spatial position by prediction boxes. It has great significance in many fields, such as face recognition, automatic driving, geographic survey, and disaster assessment. With the development of deep learning, the object detection method based on convolutional neural networks was rapidly developed in recent years, surpassing the traditional object detection methods in detection precision and speed. Usually, they are composed of Backbone, Neck, and Head. In the beginning, features of images are extracted by the Backbone then features will be aggregated by the Neck. And finally, the aggregated features will be operated by the head to predict the location and the categories of the objects present in images. Studies on the Backbone usually focus on two aspects, precision and lightweight. He et al. proposed Deep Residual Network (ResNet) [3], improved the detection performance through residual block and deeper network. A series of variants [4,5] subsequently developed based on ResNet and got better precision. Sandler proposed MobileNets [6], which works well in some lightweight object detectors. Studies on the Neck are aimed at the aggregation of features. Lin et al. proposed Feature Pyramid Networks (FPN) [7] that improve the feature extraction by aggregating the features of different layers, and He et al. proposed Spatial Pyramid Pooling (SPP) [8] that aggregates the features of different scales by processing the features separately with pooling windows of different sizes and re-fusing them after processing. In addition, some attention modules can be used as additional blocks to enhance the utilization of features in the model. Hu et al. Proposed Squeeze-and-Excitation Networks (SENet) [9], improved feature extraction by fusing features of channels. Then Efficient Channel Attention (ECA) [10] and Dilated Efficient Channel Attention (DECA) [11] were proposed based on the SENet, obtained better performance while reducing the number of parameters by introducing the convolution and dilated convolution. Recently, some detectors were proposed based on transformers [12,13]. They usually need higher equipment for training than the model based on the convolutional neural network.

The value of aerial image object detection is increasingly prominent with the increasing number of high-resolution satellites and the popularity of Unmanned Aerial Vehicles (UAV). You Only Look Once (YOLO) series detectors are suitable for aerial image object detection because of their high performance and real-time ability. They usually cluster from the training set to obtain the appropriate anchors for the subsequent training and detection. However, The Anchor Generation algorithm highly affects the detection performance of detectors because anchors can guide which layer the detector uses to detect a specific size of objects. However, the general Anchor Generation algorithm of YOLO detectors does not perform well in aerial image detection tasks as the different distribution between aerial images and nature images. Usually, the objects in aerial images are of uneven sizes and dense distribution. This uneven size distribution makes too many anchors obtained by the general algorithm try to match the majority with similar sizes and shapes. So the general Anchor Generation algorithm should be changed to be improved.

In this paper, a novel algorithm, Layered Anchor Generation (LAG), was proposed to obtain anchors from the training set and assign anchors to each layer of the object detector. The objects are divided into several layers corresponding to the number of layers of the model by diagonal size. Then anchors of each layer are gained by analyzing the aspect ratios and diagonals of the objects in the corresponding layer. In this way, the shape and size of anchors can be adjusted, respectively. Meanwhile, the anchors of each layer are no longer influenced by the overall data distribution. In summary, the contributions of this paper are as follows:

- A new Anchor Generation algorithm, LAG, was proposed. In the LAG, overfitting and mismatch problems of using the general Anchor Generation algorithm on the aerial image dataset were alleviated by dividing the objects into corresponding layers and generating anchors of each layer by analyzing objects which belong to the layer. Experiments with different input sizes and hyperparameters showed our algorithm achieves better results than the general algorithm.
- LA-YOLO method was proposed by introducing LAG into the YOLO model. The experiments under different anchor strategies have shown that the detection performance of the YOLO model was significantly improved by LAG without increasing the number of prediction boxes compared to the strategies of increasing anchors amount and adjusting the layers of anchors.
- The LAG can be used as a module with good generality for a variety of detectors. The experiments on object DetectIon in Optical Remote sensing images (DIOR) and Vision Meets Drone (VisDrone) datasets have shown that introducing LAG to other YOLO and non-YOLO detectors, such as You Only Learn One Representation (YOLOR) and Cascade Regions with CNN features (Cascade R-CNN), can significantly improve detection performance.

## 2. Related Work

### 2.1. General Object Detection

Anchors are a series of boxes with different shapes and sizes set to each layer of the detector before training. They will be scaled and offset to match the objects in the image during the training and inferring phases. The object detectors are divided into anchor-free methods and anchor-based methods according to whether the anchors are needed. Anchor-free methods include keypoint-based methods [14,15,16] and dense prediction methods [17,18,19]. The former predicts the key points of the objects, such as extreme points and centre points, and the latter regards each patch of the features as an anchor and then calculate scaling and offset for them. Anchor-based methods can be divided into one-stage methods [20,21,22,23] and two-stage methods [24,25,26], depending on whether or not to propose regions where the object may exist before detection. In one-stage methods, the features are usually extracted by Backbone, then aggregated by FPN, and finally detected by Head directly. Following the Backbone and FPN, the two-stage methods need to obtain the possible areas of objects through the Region Proposal Network (RPN), then use the Head to detect these areas.

### 2.2. Yolo Detectors

Redmon proposed YOLO [27] in 2016, which processed pictures into patches and made each patch detect objects that the center falls on. It achieved real-time object detection with a faster detection speed than two-stage detectors. In You Only Look Once version 2 (YOLOv2) [28] anchors were introduced by analyzing the training set and tiled on the patches to detectobjects by being offset and scaled. In You Only Look Once version 3 (YOLOv3) [21], the features of different layers were aggregated through the FPN structure. Objects of different sizes were detected by heads set on different layers. In You Only Look Once version 4 (YOLOV4) [29], the structure of the Cross Stage Partial Network [30] was introduced to the Backbone, and the Path Aggregation Network [31] structure was set between FPN and Heads to further aggregate features. In You Only Look Once version 5 (YOLOv5) [22], the Focus structure was introduced before the Backbone, pixel-unshuffling pictures to downsample without losing information. In addition, the label assignment strategy of the YOLO was modified in YOLOv5. Anchors of multiple patches nearby the centre of the object were regarded as positive samples to match the object during training. In YOLOR [23], the number of layers was increased, and the learning of implicit knowledge and explicit knowledge was ensembled by a unified network.

As one-stage detectors, the YOLO series detectors usually tile anchor-sized prediction boxes on each patch of the feature maps and detect objects by scaling and offsetting these prediction boxes. The heads of the model calculate a score for each prediction box, and the results of filtering by the score and the Non-Maximum Suppression (NMS) [32] algorithm are the detection results of the detectors.

### 2.3. Cascade R-CNN

Cascade R-CNN is a two-stage method commonly used in object detection. Unlike the one-stage YOLO method, it first filters a certain number of object regions that may contain objects from the anchors set in each layer of the FPN through the RPN network, then detects these regions by stacking multiple heads. In each head, samples are filtered from the previous output by a larger threshold than the former head, and then the scaling and offsets of these samples are recomputed. In this way, the model gives prediction results that keep getting closer to the labels and eventually get better detection results.

### 2.4. Aerial Image Object Detection

Aerial images are usually obtained by UAVs or satellites. Some studies on aerial image object detection have gained achievement based on the general object detectors. Rotation detector for Small, Cluttered and Rotated objects (SCRDet) [33] enhanced the features of dense objects based on Faster Regions with CNN (Faster R-CNN) [25] through pixel attention and channel attention for object detection on remote sensing images. Refined Rotation RetinaNet (R3Det) [34] was based on RetinaNet [20] and added a module to align the feature map with the object center for object detection on remote sensing images. You Only Look Twice (YOLT) [35] was based on YOLOv2 [28], increased the number of patches, and proposed the ensemble of detectors to fit the satellite remote sensing object detection. Transformer Prediction Heads-You Only Look Once version 5 (TPH-YOLOv5) [36] added Transformers, Attentions, and other improvements on YOLOv5 [22] to fit the UAV object detection. They have achieved good results, but few studies have discussed the problems of setting anchors.

### 2.5. Anchor Generation and Assignment

The anchors of YOLO detectors are generated by analyzing the training set through the K-means [37] algorithm before training. The whole process is as follows: For the model with *l* layers and *n* anchors of each layer, there are k=nl anchors in total. In the beginning, we initialize the cluster centers set $a=\{a_{1},a_{2},\cdots ,a_{k}\}$ randomly and assign each object $x_{i}$ of the training set to the nearest cluster $c_{j}$ with cluster center $a_{j}$. Next, each cluster center $a_{j}$ is recalculated by Equation (1).

(1) $$a_{j}=\frac{1}{∥c_{j}∥}\sum_{x\in c_{j}}x$$

After repeating the cluster assign and cluster center recalculate until convergence, each $a_{j}$ will be adjusted to the value that fit for the objects of the dataset. The final result of *a* includes the anchors suitable for the dataset. After sorting *a* from small to large, divide it into *l* groups and set them on heads from low layer to deep layer as the anchors of the model.

For anchor setting, some researchers proposed related methods. Zhang et al. [38] improved the matching between the anchors and the dataset by increasing the number of anchors. Ju et al. [39] suggested that anchors can be set to different layers by different scale ranges instead of evenly assigned. It improved the assignment of anchors but introduced more prediction boxes that need to be processed by the model in aerial datasets with more small objects. Hurtik et al. proposed POLY-YOLO [40], an instance segmentation model based on YOLOv3 that avoided the anchor assignment by fusing the features of different layers and then performing single-scale detection. But in this way, the structure of the model needs to be modified.

## 3. Method Introduction

### 3.1. Limitations of Anchor Generation Algorithm in YOLO

**Figure 1:**
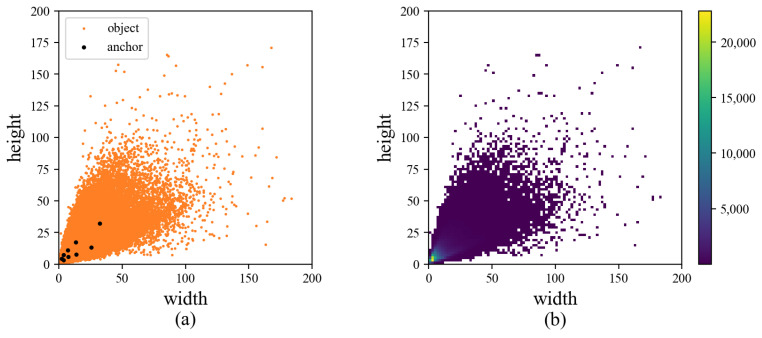
(**a**) The distribution of objects and generated anchors in the Vision Meets Drone (VisDrone) dataset. (**b**) The number of objects with different widths and heights in the VisDrone dataset.

The Anchor Generation algorithm of YOLO detectors performs a priori analysis on the training set, which can better match the anchors with the objects to be detected. But there are still some problems as follows:

- Anchors will overfit partial objects when training on datasets with uneven distribution. Obtaining anchors by the whole training set are easy to overmatch a part of objects on the dataset with uneven distribution of scale and shape. For example, there will be more anchors to match the small objects the best when most objects in the dataset are small objects, thus weakening the matching of objects of other sizes. As shown in Figure 1, the large number of small targets makes anchors prefer to match the majority.
- The anchor generation and setting are inconsistent with the idea of layered multi-scale detection. For the dataset with only small and medium objects, the anchors obtained by the whole dataset are generally small. Even the largest anchors are unsuitable for the deep layer. But the strategy of equal assigning anchors will set them to the deep layer, which results in some small objects being matched to an inappropriate head. As shown in Figure 1a, nearly all anchors obtained by the general algorithm are small, but they will still be evenly assigned from the low layer to the deep layer according to the number of heads.

### 3.2. Layered Anchor Generation

**Figure 2:**
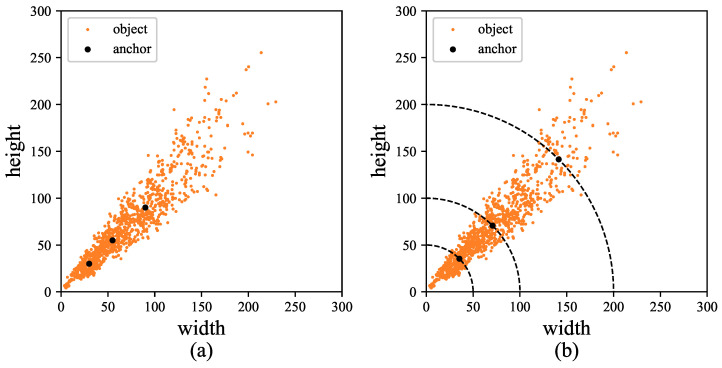
(**a**) In the general algorithm, all anchors are obtained through all objects. (**b**) In the Layered Anchor Generation (LAG), anchors of each layer are obtained by analyzing objects within the range of the layer.

To deal with the problems, the Layered Anchor Generation method is proposed inspired by the idea of divide-and-conquer. In the LAG, objects of the training set are assigned to corresponding layers according to their diagonals. Anchors of each layer are generated by matching objects within the range of this layer rather than calculated by the whole dataset as shown in Figure 2a. In this way, the generation of anchors in each layer can not be affected by the entire training set. The process is as follows: Firstly, the diagonal $d_{i}$ of each object *i* is calculated according to the width $w_{i}$ and height $h_{i}$. Then a hyperparameter *b* is introduced to get the border of each layer. Generally, the stride of the head in the latter layer is twice that of the previous layer. So, for the images with input size I∗I, the diagonal of the image is $\sqrt{2}I$, and the default diagonal of anchors in layer *j* is calculated by Equation (2).

(2) $$d_{j}=2^{j-0.5}Ib$$

Then, the anchors of each layer are matched to objects in $d_{i}\in \left(d_{j-1},d_{j+1}\right)$, as shown in Figure 2b. The matching of objects and anchors is usually determined using Intersection over Union (IoU) during the label assignment process. The IoU of objects and anchors is calculated by dividing the area of overlap between Ground Truth and anchors by the area they cover. Usually, the model selects anchors with IoU greater than a threshold *t* for each object for training, and one object may match with more than one anchor. So, in our algorithm, each layer shares a portion of the objects with other layers. Moreover, in this way, objects of the training set can be conveniently divided into *n* layers by *n* regular diagonals.

**Figure 3:**
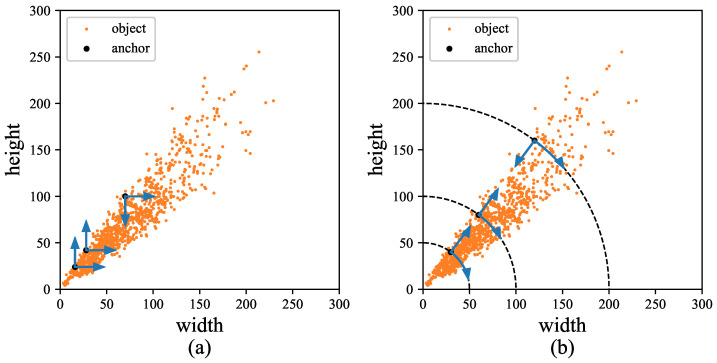
(**a**) In the general algorithm, anchors are obtained by calculating the width and height. (**b**) In the LAG, anchors are generated by calculating the aspect ratio and diagonal.

**Figure 4:**
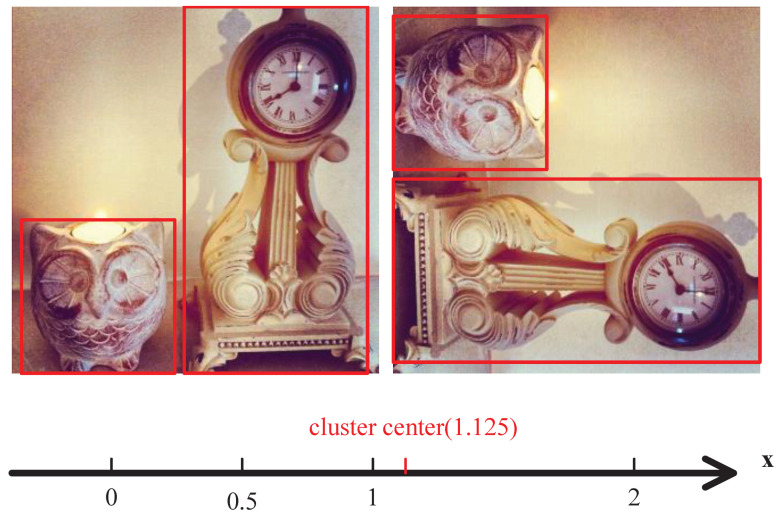
There is one object with aspect ratio of 1:2, one object with aspect ratio of 2:1, and two objects with aspect ratio of 1:1. But the aspect ratio of cluster center is 1.125:1 rather than 1:1 when clustering directly.

After layering, anchors of each layer will be calculated according to the aspect ratio and diagonal instead of width and height, as shown in Figure 3a,b. It is more suitable for the object distribution in each layer, the adjustment of the size and shape of anchors can also be decoupled in this way. If the aspect ratio r=w/h and y=h/w, there is y=1/r. There is a problem with different scales when clustering directly by *r*, objects whose height is larger than their width fall in the range (0,1) after being transformed into aspect ratios *r*, while whose width is larger than their height fall in the range (1,+∞). As shown in Figure 4, it will lead to a wrong trend that prefers the objects with r>1 when clustering directly. Therefore, the scale of *r* needs to be unified by rotating the function y=1/r by θ=π/4 using Equation (3).

(3) $$\begin{array}{c} \left\{\begin{array}{c} r^{\prime }=r\cos\left(\theta \right)-y\sin\left(\theta \right)\\ y^{\prime }=r\sin\left(\theta \right)+y\cos\left(\theta \right) \end{array}\right. \end{array}$$

After conversion, *r* and *y* are converted to $r^{\prime }$ and $y^{\prime }$, the relationship between $r^{\prime }$ and $y^{\prime }$ becomes as follows:

(4) $$\begin{array}{c} y^{\prime }=\sqrt{2+{r^{\prime }}^{2}} \end{array}$$

**Figure 5:**
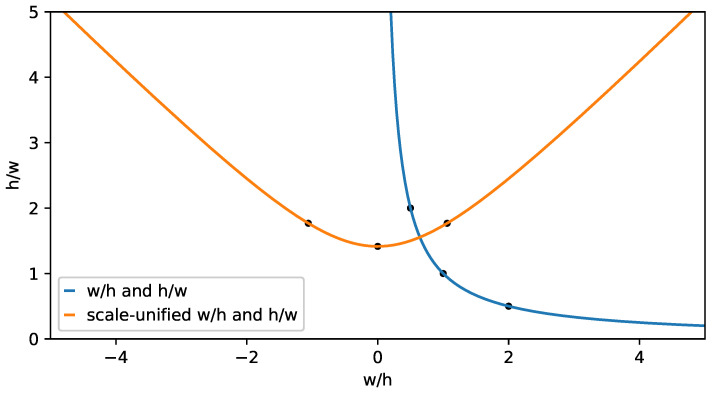
The scale of aspect ratio in different objects is unified after being handled.

Objects with r<1 and r>1 can be better clustered after converting *r* into $r^{\prime }$, as shown in Figure 5. At this time, there is:

(5) $$\begin{array}{c} r^{\prime }=\frac{\sqrt{2}}{2}\left(r-y\right) \end{array}$$

For the objects of layer *j*, the aspect ratio *r* is calculated at first and then unified to $r^{\prime }$. Next, *n* cluster centers $a_{j}^{\prime }=\left\{a_{j1}^{\prime },a_{j2}^{\prime },\cdots ,a_{jn}^{\prime }\right\}$ are gotten through the K-means algorithm by analyzing $r^{\prime }$ of these objects. After getting $a_{j}^{\prime }$, we calculate the average fitness of these cluster centers and all objects in each layer. For each object, elements which meet the the threshold condition are found from $a_{jk}^{\prime }$, and then the highest value of IoU between the object and these elements is set as the fitness of this object. Subsequently, $a_{jk}^{\prime }$ and $d_{jk}$ are fine-tuned by genetic algorithm to make them better match with the objects within the range of layer *j*, then resume the final $a_{jk}^{\prime }$ back to the aspect ratio $a_{jk}$ through Equation (3) by rotating θ=−π/4. Finally, the width and height of all anchors of layer *j* can be obtained by $a_{jk}$ and $d_{jk}$ through Equation (6).

(6) $$\begin{array}{c} \left\{\begin{array}{c} w_{jk}^{2}+h_{jk}^{2}=d_{jk}^{2}\\ w_{jk}=a_{jk}h_{jk} \end{array}\right. \end{array}$$

The pseudo-code could be found in Algorithm 1.
**Algorithm 1** Layered Anchor Generation(LAG)**Require:** 
The training set *D* with *n* objects; The threshold *t* which is used to select anchor. The list $L_{N}$ includes the number of anchors of each layer; The input size of the image, *I*; The adjustment iteration amount, *A*; The default diagonal ratio *b* of anchors in the first layer compared to the diagonal of the input size; The number of layers, *l*.**Ensure:** 
The list that includes anchors of each layer, $L_{a}$;1:Resize and fill images in *D* to *I*;2:$wh,d_{t},r$ ← Get the widths and heights list wh, diagonals list $d_{t}$, and aspect ratios list *r* from all objects in *D*;3:Build empty lists $L_{1}$, $L_{2}$, and $L_{k}$ to store the default anchor diagonals, the objects suitable for each layer, and the anchors of each layer, respectively;4:$x=\frac{\sqrt{2}}{2}\left(r-\frac{1}{r}\right)$;5:$L_{1}\left[0\right]=0$;6:**for**i=1 to *l* **do**7:    $L_{1}\left[i\right]=2^{i-0.5}Ib$;8:$L_{1}\left[l+1\right]=inf$;9:**for**i=1 to *l* **do**10:    $L_{2}\left[i\right]$ ← Select objects which diagonals are between $L_{1}\left[i-1\right]$ and $L_{1}\left[i+1\right]$ from $d_{t}$ as the matching objects of layer *i*, then obtain their width and height from wh;11:    **if** $L_{2}\left[i\right].\mathrm{length}>10L_{N}\left[i\right]$ **then**12:        *a* ← Obtain the value of $L_{N}\left[i\right]$, and then calculate corresponding cluster centers through the value of *x* corresponding to the objects in $L_{2}\left[i\right]$ using the K-means algorithm: kmeans(x[object index of $L_{2}\left[i\right]],L_{N}\left[i\right])$;13:    **else** a=[−1,0,1] // If there are few suitable objects in a layer in the dataset with extreme distribution, we can set default anchors.14:    $L_{a}\left[i\right]$ ← Expand *a* with a new dimension, set $L_{1}\left[i\right]$ as the value of the new dimension, then store to $L_{a}\left[i\right]$;15:Build an empty list $L_{f}$ to store the mean fitness of all objects in each layer;16:**for** 1 to *A* **do**17:    **for** i=1 to *l* **do**18:        $a_{wh}$ ← Get the aspect ratio $r_{a}$ from $L_{a}\left[i\right]\left[:,0\right]$, and then obtain anchors’ width and height through $r_{a}$ and $L_{a}\left[i\right]\left[:,1\right]$: $convert(L_{a}\left[i\right])$;19:        $L_{f}\left[i\right]$ ← For each object *j* in $L_{2}\left[i\right]$, select all members whose IoU is larger than *t* from $a_{wh}$. Choose the biggest member, then set the value less than 1 from magnification and minification as the fitness of object *j*. Calculate the average fitness of all objects in layer *i*: $fitness(a_{wh},L_{2}\left[i\right])$;20:        $a_{0}$←Randomly fine-tune elements in $L_{a}\left[i\right]$ and store to $a_{0}$;21:        $f_{0}=fitness\left(convert\left(a_{0}\right),L_{2}\left[i\right]\right)$;22:        **if** $f_{0}>L_{f}\left[i\right]$ **then**23:           $L_{a}\left[i\right]=a_{0}$;24:**for**i=1 to *l* **do**25:    $L_{a}\left[i\right]=convert\left(L_{a}\left[i\right]\right)$;26:**return**$L_{a}$;

## 4. Experiments and Results

### 4.1. Datasets

The effectiveness of our algorithm is tested on two public aerial image benchmarks, DIOR [41] and VisDrone [42].

**Figure 6:**
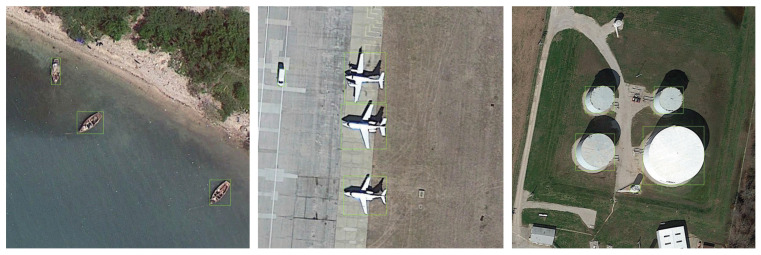
Images of the object DetectIon in Optical Remote sensing images (DIOR) dataset.

DIOR dataset is an optical remote sensing image dataset based on satellite, which contains 23,463 images and over 190,000 instances while encompassing 20 kinds of objects such as boats, dams, and stadiums. The resolution of all images in the dataset is 800 × 800. The images in DIOR are taken from an overhead view by Google Earth. Therefore, the positions of the objects in the images are relatively dispersed. Some images of the DIOR dataset are shown in Figure 6.

**Figure 7:**
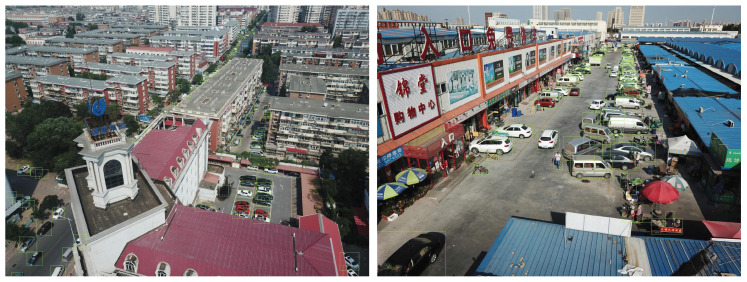
Images of the VisDrone dataset.

The VisDrone dataset is a UAV-based optical aerial image dataset that contains 10,209 images encompassing 10 types of objects, such as pedestrians, cars, and bicycles, with instances of each category average of over 50,000. The resolution of images in the dataset is up to 2000 × 1500. Images of the VisDrone dataset are captured from different viewpoints, including top-down and oblique views. As a result, objects in the images of the oblique views, especially those in the distance, will be small and relatively dense in location. Some images of the VisDrone dataset are shown in Figure 7.

### 4.2. Evaluation Criteria

The detection performance of object detectors was evaluated on criteria proposed by Microsoft in a large-scale image dataset Microsoft Common Objects in Context (MS COCO) [43], including $AP_{50}$, $AP_{75}$, $AP_{50:95}$, $AP_{S}$, $AP_{M}$, and $AP_{L}$. Among the criteria, $AP_{50}$ and $AP_{75}$ are the mean value of Average Precision(AP) of all categories under IoU = 0.5 and 0.75, respectively. $AP_{50:95}$ is the most important criterion to evaluate the performance of models. It is the mean value of AP of all categories under 10 IoU thresholds. The $AP_{S}$, $AP_{M}$, and $AP_{L}$ are the mean values of AP under all categories in small objects, medium objects, and large objects.

### 4.3. Experiment Details

The experiments were implemented on PyTorch and completed on NVIDIA Tesla V100. YOLOv3-spp, YOLOv5s, YOLOR-p6, and Cascade R-CNN were used in our experiments. In the experiments, the data augmentation and other strategies used in verifying and testing were unified as much as possible, except for some key strategies such as input resolution and model structure. The mini-batch of detectors was set to 4, and each detector of the YOLO series was trained for 200 epochs. ResNet101 [3] was used in other detectors as the backbone network. These detectors were trained for 2x epochs, using the data augment strategies that the default YOLO detectors used, such as mixup [44], mosaic [45], and photometric distortions. Other settings were left as the default public implements [22,23,45,46].

### 4.4. Ablation

#### 4.4.1. Ablation of the Hyperparameter

A hyperparameter *b* was introduced in our algorithm. It is the basis of object layering and represents the initial diagonal ratio of anchors in the lowest layer compared to the diagonal of the input image. Therefore, the value of *b* largely affects the detection effect of the model. For the detector used the strides of 8, 16, 32 of FPN to detect objects, the default diagonal ratio of anchors in the corresponding layer are *b*, 2b, and 4b respectively. Ablation experiments of hyperparameter *b* were performed on YOLOv3 to verify the impact of different hyper-parameter setting on the performance of the VisDrone dataset. The experimental results are shown in Table 1.

Experimental results have shown that the detection performance of the detector was improved by the LAG under different hyperparameter settings. When b=0.05, the model achieved the best improvement on $AP_{50}$ and $AP_{50:95}$. Other criteria also obtained improvements for varying degrees, especially $AP_{L}$ increased by 10.9. The larger we set *b*, the more improvements of the large object we get. However, when b>0.05, the detection performance of small and medium objects will gradually decrease with the increase of *b*, and the overall performance will also decrease. Therefore, when b=0.05, our algorithm obtains optimal anchors for each head. *b* can be reduced for datasets with mainly small objects and increased for datasets with majorly large objects.

#### 4.4.2. Ablation of the Input Size

The receptive field of each layer is not affected by the input size of the model, but the change of input size impacts the result of the Anchor Generation algorithm, thus affecting the matching effect between anchors and layers. On the one hand, the degree of feature loss after convolutions is different for images with different input sizes. On the other hand, the number of the prediction boxes in each layer is different under different input sizes. So the effects of the generated anchors are impacted by the input size. Ablation experiments of input sizes were performed on YOLOv3 to test the performance of our algorithm at different input sizes on VisDrone, and the results were shown in Table 2.

Our method achieved the best improvement under the input size 416×416 on YOLOv3. Meanwhile, it can still obtain good results on other input sizes. Under four kinds of input size, considerable improvement was obtained on the YOLOv3 model on VisDrone.

### 4.5. Experiments on Different Anchor Settings

To verify the head of each layer in the YOLO model has its suitable detection range, some experiments with different anchor settings were conducted. Because the amount of prediction boxes in different layers is different, the layer of anchors and the number of anchors in each layer will affect detectors. But few studies explored this topic. In the general algorithm, the amount of anchors in each head is the same, and 3 anchors will be assigned to each layer. So we obtained 9 anchors on the DIOR dataset based on the general algorithm and assigned 3 to each layer head from the lower to the deeper for experimentation, expressed as [3,3,3]. According to this expression, experiments in other anchor settings were also implemented, such as [9,0,0], [0,9,0], [0,0,9], [4,2,3], and so on. Meanwhile, an experiment with 12 Anchors based on the general algorithm was also performed, expressed as [4,4,4]. The setting of anchors affects the number of prediction boxes to be processed by the model. The calculation equation is Equation (7), where *I* is the input size, $n_{i}$ is the number of anchors of layer *i*, and $s_{i}$ is the stride of the corresponding layer.

(7) $$\begin{array}{c} p_{i}=\sum_{i}\frac{n_{i}I^{2}}{s_{i}^{2}} \end{array}$$

The data in Table 3 showed the detection performance of the detector under different anchor settings. In experiment [4,4,4], the detection performance of small and medium objects was improved by increasing the number of anchors. However, the improvement reflected in the detection results of overall objects is not significant, and more prediction boxes need to be processed. In experiment [0,0,9], the detection performance was limited by too few prediction boxes, but increased prediction boxes do not certainly bring better detection performance. It could be found that the number of prediction boxes has approximately doubled by comparing the results of experiments [9,0,0] and [3,3,3], but the detection performance declined. Since only single layer was used for detection in experiments [9,0,0], [0,9,0], [0,0,9], experiments on anchor settings of [7,1,1], [1,7,1] and [1,1,7] were also conducted to detect objects by multi-layers. From the results, it could be found that the strategy of dividing anchors into multi-layers to detect improves the detection performance significantly. But even if the same anchors setting in different layers, the impact on the results is still very diverse. In experiment [7,1,1], the number of prediction boxes was the most, but the detection performance was not better than that of evenly assigned anchors. However, in the experiment [4,2,3], the detector obtained better results with fewer prediction boxes than the results of experiment [4,4,4] by adjusting the layer of anchors. Therefore, more anchors are not necessarily better. There is a suitable detection range in each layer, and matching anchors with the detection range of the layer when the number of bounding boxes is sufficient can improve the detection performance more effectively than increasing the number of prediction boxes.

We compared LAG with other anchor strategies, such as adjusting the layer of anchors and increasing the number of anchors. To further complement and comparison, experiments under more anchors were conducted. In addition, by analyzing the anchors and the ranges given in related literature [39], anchors were adjusted to [6,1,2] to experiment. The data in Table 4 are the results. The strategy of adjusting the layer of anchors improved the detection performance, but the maximum improvement was not as great as increasing the number of anchors in each layer. The detector obtained better results by increasing the number of anchors until the number of anchors in each layer was increased to 6. At this point, the results of increasing the number of anchors have exceeded adjusting the layer of anchors, but the number of prediction boxes that the detector needs to process also reached twice the general state. The detection performance was decreased if the anchors continued to increase. Anchors, objects, and the detector were correlated in the LAG. Compared with adjusting the layer of anchors, LAG matched anchors with the suitable range of each layer at the time of generation, obtaining better results without changing the number of prediction boxes in each layer. Compared with increasing the number of anchors, LAG matched the objects to layers of the detector, thus better matching the dataset to the detector with fewer anchors. Our method obtained better results using fewer prediction boxes.

In addition, a phenomenon could be found in the experiment [4,4,4]. The detection performance of small and medium objects was significantly improved, but of all objects was not. For this phenomenon, the detection performance of each category was examined one by one by COCOAPI. The data in Table 5 were the results. In the table, “None” meant that there were no objects of this category belonging to this scale, while “0” meant that the detector failed to detect objects of this category belonging to this scale. It could be found that the small, medium, and large objects were different impacts on the overall detection performance because of the different proportions of the small, medium, and large objects in each category. For example, most of the objects belonging to the “dam” category were large objects, but small and medium objects were over-emphasized so that the overall detection performance decreased. Another example was that the reduction of detection performance of large objects results in no significant degradation of the overall detection performance on the “ship” category because of the small number of large objects.

### 4.6. Effects on Different Detectors

LAG was used in multiple detectors of the YOLO series and tested on different aerial datasets to verify the generality of our algorithm. At the same time, it was used on Cascade R-CNN [26] to test if it could be used in the non-YOLO detector. The experiment results on the DIOR and VisDrone datasets were shown in Table 6 and Table 7, respectively. The input size strategies of YOLO series detectors were different from other detectors. The images were usually scaled to a uniform size in the YOLO detectors. The input size of Cascade R-CNN in the tables meant dividing the long side of the image by 1333 and the short side by 800 and taking the smaller value as the image scaling factor. Compared with the general Anchor Generation algorithm, more improvement was demonstrated by the LAG on VisDrone than on DIOR. For this phenomenon, we analyzed the size of objects in the DIOR and VisDrone datasets, the proportions of the small, medium, and large objects of COCO standard in the DIOR dataset are around 42%, 29%, and 29%, and these proportions in VisDrone are around 60%, 34%, and6%. The uneven object distribution aggravated the overfitting of some objects by the general Anchor Generation algorithm, bug it could be alleviated by LAG.

It could be found from the results of the tables that our algorithm achieves positive results in a variety of YOLO models. In addition, our algorithm also leads to a significant improvement in detection performance on the non-YOLO model CascadeRCNN. It proves there is a good generality in the LAG on various detectors.

### 4.7. Compare with Other Sota Detectors

LA-YOLOv3, LA-YOLOv5, LA-YOLOR, and LA-Cascade R-CNN are the methods that introduced LAG. They were compared with other SOTA detectors on DIOR and VisDrone datasets to demonstrate the high performance of the detectors added our method. These detectors are AutoAssign [47], Neural Architecture Search-Fully Convolutional One-Stage object detector (NAS-FCOS) [48], Probabilistic Anchor Assignment(PAA) [49], VarifocalNet [50], and Adaptive Training Sample Selection (ATSS) [51]. The data in Table 8 are theexperiment results on the DIOR dataset and in Table 9 are the experiment results on the VisDrone dataset. LA-YOLOv3 and LA-YOLOv5 were better on the DIOR dataset but worse on the VisDrone dataset than most detectors. The larger input size made the detector more likely to learn better features. But a larger input size could also lead to a reduction in detection speed. The real-time performance of the YOLO detectors was also related to some extent to the input size strategy. The difference in input size between the YOLO detectors and the others caused a different degrees of mpact on various datasets. It can be seen from the results in the tables that the detectors added the LAG demonstrated competitive results on the DIOR and VisDrone datasets.

It can be seen from the results of the tables that LA-YOLOR shows the best results in both the DIOR and VisDrone datasets, and other methods which we proposed also obtained competitive results.

## 5. Discussion

The problems of the general anchor generation method on the optical aerial datasets were alleviated by LAG from two aspects. On the one hand, anchors of different layers were more susceptible to being affected by partial objects when anchors of each layer were generated by all the objects together. In the LAG, the objects were layered by their diagonals. Anchors of each layer were just affected by objects within the corresponding range. On the other hand, anchors were assigned to inappropriate layers because of the equal assignment. In the LAG, anchors were matched with the detection range of each layer of the model. In this way, the anchors of each layer were suitable for the layer.

Compared with other anchor strategies, such as adjusting the layer of anchors and increasing the number of anchors, introducing the LAG in the detectors obtained better results without changing the number of anchors and the number of prediction boxes. However, there are still some demerits in the LAG. First, an additional hyperparameter is introduced to calculate the boundaries of each layer. We have tried to replace this hyperparameter by clustering the boundaries of each layer, but the results were not satisfactory. Second, the effectiveness of the anchor strategy is strongly influenced by the distribution of the dataset. As one of the strategies used in object detectors, LAG can be introduced into detectors and then applied to other fields, but its effectiveness can be affected by many factors, such as different data distribution and different features learned by the models. We will continue to explore how to improve LAG to achieve a no-hyperparameter algorithm and better adapt it to more domains.

## 6. Conclusions

The performance of the anchor-based detector depends heavily on the setting of the anchor, objects of different sizes will be detected by different layers according to the anchor settings. However, the Anchor Generation algorithm of YOLO detectors can not adapt to the extreme distribution of aerial images well. It can be found by analyzing objects in the aerial image dataset that the anchors generated by the general algorithm for training suffer from overfitting because of the large number of small objects. Meanwhile, all anchors are obtained directly based on all objects, but they are divided equally to each layer in the assignment, which leads to anchors being assigned to suboptimal layers.

In this paper, LAG was proposed to alleviate the problems of the general Anchor generation algorithm on the optical aerial image datasets by introducing the idea of divide-and-conquer. Objects were layered at first, and then anchors of each layer were generated by analyzing the objects of the layer separately. Experiments under different conditions showed that our method has a good generality. Using our method on both YOLO and non-YOLO models got good results, the detectors such as LA-YOLOR and LA-Cascade R-CNN with the introduction of LAG achieved competitive results on the optical aerial image datasets DIOR and VisDrone. In addition, since the structure of the detectors was not modified and the number of prediction boxes was not changed, the improvement obtained by the LAG was cost-free. 

## Figures and Tables

**Table 1:** Ablation results on different hyperparameter.

Method	Input Size	${\mathrm{AP}}_{50}$	${\mathrm{AP}}_{75}$	${\mathrm{AP}}_{S}$	${\mathrm{AP}}_{M}$	${\mathrm{AP}}_{L}$	${\mathrm{AP}}_{50:95}$
YOLOv3	(416,416)	31.2	15.1	9.4	24.4	31.2	16.2
+LAG(d = 0.025)		32.1	16.7	9.3	26.7	38.7	17.5
+LAG(d = 0.05)		**33.7**	**17.8**	**9.8**	**28.5**	42.1	**18.7**
+LAG(d = 0.1)		33.0	17.1	9.1	27.7	42.9	18.1
+LAG(d = 0.125)		32.9	16.6	9.1	27.1	43.0	17.8
+LAG(d = 0.15)		32.2	16.5	8.7	26.3	**44.8**	17.5
+LAG(d = 0.2)		32.6	16.2	8.9	26.1	43.7	17.4

**Table 2:** Ablation results on different input size.

Method	Input Size	${\mathrm{AP}}_{50}$	${\mathrm{AP}}_{75}$	${\mathrm{AP}}_{S}$	${\mathrm{AP}}_{M}$	${\mathrm{AP}}_{L}$	${\mathrm{AP}}_{50:95}$
YOLOv3	(320,320)	23.6	10.4	6.4	17.9	28.4	11.8
+LAG		26.2	13.0	6.3	21.9	36.6	14.0
YOLOv3	(416,416)	31.2	15.1	9.4	24.4	31.2	16.2
+LAG		33.7	17.8	9.8	28.5	42.1	18.7
YOLOv3	(640,640)	42.8	22.6	15.5	34.1	43.7	24.0
+LAG		45.0	25.5	16.2	37.9	50.1	26.0
YOLOv3	(768,768)	47.2	26.4	18.5	37.2	51.0	27.0
+LAG		48.4	28.2	18.8	39.8	53.1	28.2

**Table 3:** Experiment results under different anchor settings on the DIOR dataset.

Method	Input Size	Settings ^1^	${\mathrm{AP}}_{50}$	${\mathrm{AP}}_{75}$	${\mathrm{AP}}_{S}$	${\mathrm{AP}}_{M}$	${\mathrm{AP}}_{L}$	${\mathrm{AP}}_{50:95}$	Quantities ^2^
YOLOv3	(416,416)	[3,3,3]	79.9	63.0	8.6	36.3	71.9	58.6	10,647
		[9,0,0]	77.8	60.8	8.8	35.9	70.1	56.0	24,336
		[7,1,1]	79.0	62.7	8.0	34.4	71.6	58.1	19,773
		[0,9,0]	77.7	61.2	7.5	36.9	70.9	56.7	6084
		[1,7,1]	78.9	62.0	8.7	33.9	70.7	57.6	7605
		[0,0,9]	74.6	58.2	4.2	31.9	69.8	54.3	1521
		[1,1,7]	78.6	59.3	8.9	33.9	67.9	55.3	4563
		[4,2,3]	80.2	63.7	8.4	37.8	72.3	59.3	12,675
		[4,4,4]	79.7	63.1	10.2	37.6	71.9	58.7	14,196

^1^ The amount of anchors setting on each layer from low to deep. ^2^ The Quantities of prediction boxes that the detector needs to process on each setting.

**Table 4:** Experiment results under different anchor strategies on the DIOR dataset.

Method	Input Size	Settings	${\mathrm{AP}}_{50}$	${\mathrm{AP}}_{75}$	${\mathrm{AP}}_{S}$	${\mathrm{AP}}_{M}$	${\mathrm{AP}}_{L}$	${\mathrm{AP}}_{50:95}$	Quantities
YOLOv3	(416,416)	[3,3,3]	79.9	63.0	8.6	36.3	71.9	58.6	10,647
		[4,2,3]	80.2	63.7	8.4	**37.8**	72.3	59.3	12,675
		[6,1,2]	80.0	63.7	8.8	36.2	72.5	59.2	17,238
		[4,4,4]	79.7	63.1	10.2	37.6	71.9	58.7	14,196
		[5,5,5]	80.0	63.4	9.3	37.2	72.3	59.2	17,745
		[6,6,6]	80.4	64.1	**10.7**	37.4	72.4	59.6	21,294
		[7,7,7]	80.0	63.5	8.6	37.7	72.4	59.2	24,843
		[8,8,8]	80.3	63.9	8.9	37.0	72.6	59.5	28,392
		[9,9,9]	80.2	63.6	9.2	37.0	72.6	59.4	31,941
		[3,3,3]+LAG	**80.8**	**64.2**	9.0	36.9	**73.1**	**59.8**	10,647

**Table 5:** The detection performance of each category under different anchor settings.

Category	[3,3,3]				[4,4,4]				[3,3,3]+LAG			
	${\mathrm{AP}}_{S}$	${\mathrm{AP}}_{M}$	${\mathrm{AP}}_{L}$	${\mathrm{AP}}_{50:95}$	${\mathrm{AP}}_{S}$	${\mathrm{AP}}_{M}$	${\mathrm{AP}}_{L}$	${\mathrm{AP}}_{50:95}$	${\mathrm{AP}}_{S}$	${\mathrm{AP}}_{M}$	${\mathrm{AP}}_{L}$	${\mathrm{AP}}_{50:95}$
airplane	6.5	47.0	88.3	80.6	11.8	49.1	88.1	80.9	10.1	48.6	88.6	81.1
airport	None	15.2	59.7	58.1	None	17.7	59.8	58.4	None	12.6	61.3	59.6
baseballfield	7.7	77.9	89.4	83.6	6.3	75.8	89.3	83.1	6.7	78.9	89.6	83.7
basketballcourt	0	52.4	86.8	75.2	0	54.3	87.2	75.9	0	52.9	86.9	75.0
bridge	4.7	19.2	59.4	32.6	5.4	16.3	59.3	32.1	5.0	19.3	61.5	34.3
chimney	0	2.6	86.9	81.1	0	5.7	87.1	81.2	0	6.7	87.5	82.0
dam	3.1	19.3	39.9	36.5	20.9	21.7	39.1	36.2	0.7	25.2	43.2	39.7
Expressway-Service-area	0	3.3	73.9	57.2	0	3.9	73.1	57.2	0	7.2	75.6	60.4
Expressway-toll-station	5.5	60.2	87.2	57.1	5.4	58.4	86.5	56.0	9.7	61.7	88.5	59.5
golffield	None	7.8	60.6	58.3	None	7.1	61.8	59.6	None	6.6	64.1	61.6
groundtrackfield	17.6	46.1	85.8	65.3	19.9	46.2	85.1	65.4	18.5	46.5	85.3	65.3
harbor	4.1	17.5	55.8	46.5	5.2	19.5	55.7	46.5	3.8	21.3	56.6	47.9
overpass	2.1	14.1	66.9	44.9	3.0	15.2	67.5	45.5	3.5	14.2	69.1	47.2
ship	39.6	62.6	73.3	49.5	39.4	62.8	72.8	49.7	39.5	62.3	72.8	49.3
stadium	0	2.5	75.2	73.4	0.2	21.5	74.8	73.0	0	2.6	78.5	76.7
storagetank	9.5	75.2	89.9	70.1	9.7	75.7	89.7	70.3	8.9	76.4	90.2	70.4
tenniscourt	6.4	62.4	96.1	83.6	6.4	62.5	96.3	83.9	8.0	62.7	96.5	84.2
trainstation	0	18.2	31.0	29.7	0	14.8	31.5	30.0	0	12.9	33.5	31.8
vehicle	32.6	69.3	62.4	47.8	32.5	69.4	63.7	47.8	31.6	68.4	63.1	46.5
windmill	16.3	52.7	68.6	40.7	17.3	53.7	70.5	41.8	15.3	51.7	70.0	39.9
mean	8.6	36.3	71.9	58.6	10.2	37.6	71.9	58.7	9.0	36.9	73.1	59.8

**Table 6:** The effects of LAG on different detectors under the DIOR dataset.

Method	Input Size	${\mathrm{AP}}_{50}$	${\mathrm{AP}}_{75}$	${\mathrm{AP}}_{S}$	${\mathrm{AP}}_{M}$	${\mathrm{AP}}_{L}$	${\mathrm{AP}}_{50:95}$
YOLOv3	(416,416)	79.9	63.0	8.6	36.3	71.9	58.6
+LAG		80.8	64.2	9.0	36.9	73.1	59.8
YOLOv5	(640,640)	79.8	62.5	9.1	38.4	69.5	57.5
+LAG		80.2	63.1	8.7	38.4	70.9	58.4
YOLOR	(1280,1280)	80.6	66.5	14.1	40.3	75.0	62.4
+LAG		80.9	67.7	13.8	40.6	75.8	63.0
Cascade R-CNN	(1333|800)	74.9	60.2	7.2	32.5	66.9	54.4
+LAG		75.6	60.9	7.7	33.1	68.0	55.3

**Table 7:** The effects of LAG on different detectors under the VisDrone dataset.

Method	Input Size	${\mathrm{AP}}_{50}$	${\mathrm{AP}}_{75}$	${\mathrm{AP}}_{S}$	${\mathrm{AP}}_{M}$	${\mathrm{AP}}_{L}$	${\mathrm{AP}}_{50:95}$
YOLOv3	(416,416)	31.2	15.1	9.4	24.4	31.2	16.2
+LAG		33.7	17.8	9.8	28.5	42.1	18.7
YOLOv5	(640,640)	33.0	17.4	10.2	27.4	36.4	18.2
+LAG		34.7	19.6	10.7	30.5	42.9	19.9
YOLOR	(1280,1280)	54.9	35.6	25.3	46.0	55.7	34.4
+LAG		57.0	37.8	26.6	49.3	59.3	36.2
Cascade R-CNN	(1333|800)	38.8	24.7	14.3	35.7	43.4	23.7
+LAG		42.3	25.8	17.0	35.0	41.3	25.2

**Table 8:** The detection performance of different detectors on the DIOR dataset.

Method	Input Size	${\mathrm{AP}}_{50}$	${\mathrm{AP}}_{75}$	${\mathrm{AP}}_{S}$	${\mathrm{AP}}_{M}$	${\mathrm{AP}}_{L}$	${\mathrm{AP}}_{50:95}$
AutoAssign	(1333|800)	76.7	56.4	7.5	32.0	64.1	52.2
NAS-FCOS	(1333|800)	72.8	51.3	6.0	29.4	59.5	48.1
PAA	(1333|800)	74.8	57.0	6.0	31.1	65.9	52.9
VarifocalNet	(1333|800)	74.0	57.9	6.5	32.4	66.3	53.3
ATSS	(1333|800)	73.5	56.3	6.4	31.5	64.2	51.9
LA-YOLOv3	(416,416)	80.8	64.2	9.0	36.9	73.1	59.8
LA-YOLOv5	(640,640)	80.2	63.1	8.7	38.4	70.9	58.4
LA-YOLOR	(1280,1280)	80.9	67.7	13.8	40.6	75.8	63.0
LA-Cascade R-CNN	(1333|800)	75.6	60.9	7.7	33.1	68.0	55.3

**Table 9:** The detection performance of different detectors on the VisDrone dataset.

Method	Input Size	${\mathrm{AP}}_{50}$	${\mathrm{AP}}_{75}$	${\mathrm{AP}}_{S}$	${\mathrm{AP}}_{M}$	${\mathrm{AP}}_{L}$	${\mathrm{AP}}_{50:95}$
AutoAssign	(1333|800)	36.2	19.8	12.4	29.8	37.4	20.4
NAS-FCOS	(1333|800)	31.5	18.0	9.8	28.1	36.4	18.1
PAA	(1333|800)	36.0	21.3	11.4	32.8	44.4	21.1
VarifocalNet	(1333|800)	38.0	23.6	13.8	34.1	42.8	22.8
ATSS	(1333|800)	36.8	22.0	12.6	33.7	42.3	21.8
LA-YOLOv3	(416,416)	33.7	17.8	9.8	28.5	42.1	18.7
LA-YOLOv5	(640,640)	34.7	19.6	10.7	30.5	42.9	19.9
LA-YOLOR	(1280,1280)	57.0	37.8	26.6	49.3	59.3	36.2
LA-Cascade R-CNN	(1333|800)	42.3	25.8	17.0	35.0	41.3	25.2

## Data Availability

The datasets used in this study are all public datasets.

## Abbreviations

The following abbreviations are used in this manuscript:

YOLO	You Only Look Once
LAG	Layered Anchor Generation
YOLOv3	You Only Look Once version 3
YOLOv5	You Only Look Once version 5
YOLOR	You Only Learn One Representation
Cascade R-CNN	Cascade Regions with CNN features
VisDrone	Vision Meets Drone
DIOR	object DetectIon in Optical Remote sensing images
ResNet	Deep Residual Network
FPN	Feature Pyramid Networks
SPP	Spatial Pyramid Pooling
SENet	Squeeze-and-Excitation Networks
ECA	Efficient Channel Attention
DECA	Dilated Efficient Channel Attention
UAV	Unmanned Aerial Vehicles
RPN	Region Proposal Network
YOLOv2	You Only Look Once version 2
YOLOv4	You Only Look Once version 4
NMS	Non-Maximum Suppression
SCRDet	Rotation detector for Small, Cluttered and Rotated objects
Faster R-CNN	Faster Regions with CNN
R3Det	Refined Rotation RetinaNet
YOLT	You Only Look Twice
TPH-YOLOv5	Transformer Prediction Heads-You Only Look Once version 5
IoU	Intersection over Union
MS COCO	Microsoft Common Objects in Context
AP	Average Precision
NAS-FCOS	Neural Architecture Search-Fully Convolutional One-Stage object detector
PAA	Probabilistic Anchor Assignment
ATSS	Adaptive Training Sample Selection
